# Dental Composites with Magnesium Doped Zinc Oxide Nanoparticles Prevent Secondary Caries in the Alloxan-Induced Diabetic Model

**DOI:** 10.3390/ijms232415926

**Published:** 2022-12-14

**Authors:** Tahreem Tanweer, Nosheen Fatima Rana, Iqra Saleem, Iqra Shafique, Sultan M. Alshahrani, Hanadi A. Almukhlifi, Amenah S. Alotaibi, Sohad Abdulkaleg Alshareef, Farid Menaa

**Affiliations:** 1 Department of Biomedical Engineering and Sciences, School of Mechanical & Manufacturing Engineering, National University of Science & Technology (NUST), Islamabad 44000, Pakistan; 2 Clinical Pharmacy Department, College of Pharmacy, King Khalid University, Abha 61441, Saudi Arabia; 3 Department of Chemistry, Faculty of Science, University of Tabuk, Tabuk 71491, Saudi Arabia; 4 Genomic and Biotechnology Unit, Department of Biology, Faculty of Science, University of Tabuk, Tabuk 71491, Saudi Arabia; 5 Department of Chemistry, College of Duba, University of Tabuk, Tabuk 71491, Saudi Arabia; 6 Departments of Internal Medicine and Nanomedicine, California Innovations Corporation, San Diego, CA 92037, USA

**Keywords:** dental materials, nanomaterials, oral biofilm, antibacterial, secondary caries

## Abstract

Antibacterial restorative materials against caries-causing bacteria are highly preferred among high-risk patients, such as the elderly, and patients with metabolic diseases such as diabetes. This study aimed to enhance the antibacterial potential of resin composite with Magnesium-doped Zinc oxide (Mg-doped ZnO) nanoparticles (NPs) and to look for their effectiveness in the alloxan-induced diabetic model. Hexagonal Mg-doped ZnO NPs (22.3 nm diameter) were synthesized by co-precipitation method and characterized through ultraviolet-visible (UV-Vis), Fourier transform infrared (FTIR) spectroscopy, X-ray diffraction (XRD), scanning electron microscopy (SEM), and energy dispersive spectroscopy (EDS) analysis. The Mg-doped ZnO NPs (1, 2.5 and 5% *w*/*w*) were then evaluated for antibacterial activity using a closed system in vitro biofilm model. Significant enhancement in the antibacterial properties was observed in composites with 1% Mg-doped ZnO compared to composites with bare ZnO reinforced NPs *(Streptococcus mutans*, *p* = 0.0005; *Enterococcus faecalis*, *p* = 0.0074, Saliva microcosm, *p* < 0.0001; Diabetic Saliva microcosm, *p* < 0.0001). At 1–2.5% Mg-doped ZnO NPs concentration, compressive strength and biocompatibility of composites were not affected. The pH buffering effect was also achieved at these concentrations, hence not allowing optimal conditions for the anaerobic bacteria to grow. Furthermore, composites with Mg-doped ZnO prevented secondary caries formation in the secondary caries model of alloxan-induced diabetes. Therefore, Mg-doped ZnO NPs are highly recommended as an antibacterial agent for resin composites to avoid biofilm and subsequent secondary caries formation in high-risk patients.

## 1. Introduction

Secondary caries (SC) formation around dental resin composites is the major cause of restoration failure [1,2]. It is also a common oral manifestation among adults and patients with metabolic disorders such as diabetes [3]. Multiple risk factors are reported as the main cause, such as polymerization shrinkage and subsequent bacterial microleakage due to restoration–tooth interface debonding and higher plaque accumulation [1,2,3,4,5]. The oral microbiome is switched towards acid-producing bacteria, lowering the pH and causing mineral loss [6]. Multiple risk factors are involved in SC formation, such as type of restoration, patient’s caries risk, class of restoration, salivary flow rate, poor hygiene, unhealthy eating habits, etc. [2,3,4,5]. Among patient characteristics, the risk of SC is even higher among patients with metabolic disorders, such as diabetic patients [5]. Diabetes alters the oral environment by modifying salivary pH, which affects the resin composites’ surface roughness, allowing more plaque to build up on the restoration surface, and resin-tooth bond weakening [5,6]. Due to low pH, salivary flow reduces, causing a low acid buffering effect. Oral microflora shifts towards anaerobic biofilm-making bacteria [5,6,7]. It is also known that diabetics exhibit higher levels of oral anaerobic bacteria compared to those without diabetes [7,8]. Apart from patient characteristics, restoration characteristics are also responsible for SC [8]. The most used and preferred restoration type, i.e., resin composite, is more prone to biofilms compared to amalgam [4]. There is a high need for composites with enhanced antibacterial properties, and acid-buffering effects are highly desired to avoid SC in high-risk individuals [2].

Nanotechnology is a pioneering concept in manufacturing dental materials with enhanced characteristics and antimicrobial properties [8]. Several studies have reported the application of antibacterial nanoparticles (NPs) in multiple applications, such as in biomedicine, dentistry, agriculture, etc. [9,10,11]. Special interest has been centered on inorganic nanocrystalline metal oxides to achieve this, as they exhibit exceptionally high surface area-to-volume ratios and are highly suitable in restorative applications [9,10,11,12]. They show superior durability and less toxicity compared to their counterparts [13,14]. One such inorganic metal oxide is Zinc oxide (ZnO), which is used as an opaque filler material in the resin matrix [10,12]. It is a highly abundant, inexpensive material, categorized as a generally recognized as safe (GRAS) material by the Food and Drug Administration [15], making it suitable for many biomedical applications. Furthermore, its longer durability makes it superior to other dental filler materials [13]. It is also not toxic to human cells at low concentrations (up to >40 μg/mL) and exhibits a strong bacterial growth-inhibiting characteristic [16,17]. It also exhibits better antimicrobial activity against bacteria that contributes to dental caries formation, such as *S. Mutans* [11]. When incorporated into the resin, it also does not negatively affect the aesthetics of the resin composites [11,12,13]. However, composites with ZnO NPs have limited antibacterial properties at lower concentrations (1% by weight) and against multi-species biofilms [10], whereas increasing the concentration of ZnO NPs negatively affects the mechanical properties of the resin composite [18]. Hence, additional research must be carried out to make it more attractive.

Doping is a powerful approach to augment the physiochemical properties of ZnO [19,20]. Doping ZnO reduces the electron/hole recombination, increases the surface area to volume ratio, and improves stability towards dissolution [21]. Transition metals such as Gold (Au) [22], Silver (Ag) [23], Copper (Cu) [24], Iron (Fe) [25], and rare earth metals such as Calcium (Ca) [26], etc. have been previously utilized to dope ZnO. Evidence suggests that doped ZnO NPs exhibit enhanced antibacterial properties with respect to their pure material counterpart [23]. Doped ZnO has been used in multiple applications, such as in the eradication of bacteria for water purification as well as in preventing biofilm formation [19,27]. There is limited evidence that reported the application of doped ZnO in dentistry for the eradication of oral infection-causing bacteria.

Mg is a low-density shiny silvery-white rare earth metal with an elastic modulus like cortical bone (E = 45 GPa) [28,29]. It exhibits significant antibacterial activity and impairs bacterial adhesion and biofilm formation, making it less susceptible to adverse conditions [30]. In dentistry, it plays a significant role in preventing caries and periodontal disease due to its distinctive capacity to reduce inflammation caused by bacterial toxins. Furthermore, it has been reported to enhance the pH from acidic to physiological pH [31,32,33]. There is limited research that reports the effectiveness of Mg-doped ZnO NPs in enhancing the antibacterial activity of resin composites against caries-causing bacteria in the natural oral environment.

Therefore, the present study aims to synthesize and characterize Mg-doped ZnO NPs and the addition of these NPs in resin composite restorative materials to ameliorate their antibacterial activity without negatively affecting the mechanical properties, biocompatibility, and aesthetics. Furthermore, we tested these composites in a preclinical study using an animal model of secondary caries in alloxan-induced diabetic rats [32]. The CARS criteria for SC assessment were utilized for the first time in the diabetic model of SC.

## 2. Results

### 2.1. Successful Synthesis of Bare and Mg-Doped ZnO NPs

**Figure 1:**
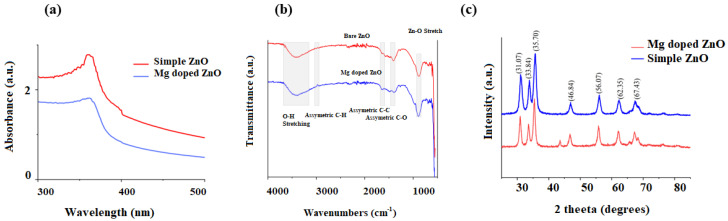
(**a**) UV-vis analysis, (**b**) FTIR analysis, and (**c**) XRD analysis of bare and Mg-doped ZnO NPs.

The bare and Mg-doped ZnO NPs were successfully prepared using the coprecipitation method. The UV peak of bare ZnO NPs was obtained at 362 nm, confirming its synthesis. Upon doping with Mg, no change in precipitate color was observed. The Uv shift from 362 to 358 nm, i.e., the blue shift, confirmed the doping of ZnO with Mg [34]. (Figure 1a).

### 2.2. FTIR Analysis of Bare and Mg-Doped ZnO NPs

A broad absorption band, referring to the OH stretching of water, Zn–OH, and Mg–Zn–OH, was observed at 3600–3000 cm^−1^ [34,35]. The peaks at 2971 and 2974 for bare and Mg-doped ZnO NPs showed asymmetric C–H bonds. The C=C stretching was seen at 1619 and 1605 cm^−1^ for bare and doped ZnO NPs. The C=O symmetric stretching vibration was detected at 1459 and 1438 cm^−1^ for bare and Mg-doped samples, respectively. Weak stretching frequencies of Zn–O were seen at 886 and 874 cm^−1^ for bare and doped ZnO NPs samples [34,35,36,37] (Figure 1b).

### 2.3. XRD Analysis of Bare and Mg-Doped ZnO NPs

The XRD patterns for the bare and Mg-doped ZnO are presented in Figure 1c. Major peaks were observed at 31.07, 33.84, 35.70, 46.84, 56.07, 62.35, and 67.43. The XRD pattern shows the wurtzite-like hexagonal structure of bare and Mg-doped ZnO NPs [35]. The two theta values corresponded to the JCPDS card number 79-2205 [34]. Broader peaks and lower intensities of Mg-doped ZnO NPs patterns compared to bare ZnO confirmed the successful doping of Mg on the ZnO crystal lattice [35,36]. The doping was favored because of the small ionic radius of Mg^2+^ compared to Zn^2+^ [36]. Using the XRD patterns, the Mg-doped ZnO NP’s crystal size was determined using the Scherer equation.

$$D=\frac{k\lambda }{\beta \mathrm{hkl}\;\cos\;\theta },$$

where k is the constant with a value of 0.90. λ refers to the wavelength of incidence X-ray (0.154) nm. βhkl refers to the full width at half maximum, whereas cos θ denotes the peak’s position.

### 2.4. SEM and EDX of Bare and Mg-Doped ZnO

**Figure 2:**
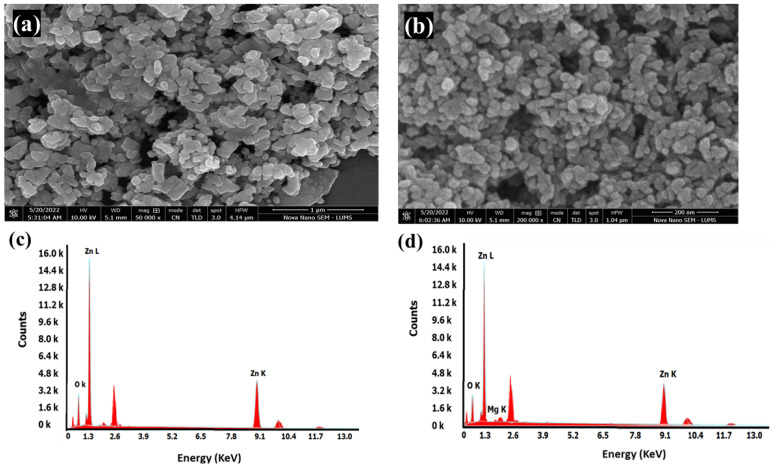
(**a**) SEM of Bare ZnO NPs, (**b**) SEM of Mg-doped ZnO, (**c**) EDX of Bare ZnO NPs, and (**d**) EDX of Mg-doped ZnO NPs.

The morphology of the bare and Mg-doped ZnO NPs is presented in Figure 2. The average particle size was calculated using Image J software. Hexagonal-shaped ZnO NPs and Mg-doped ZnO NPs were obtained with particle sizes of 25.87 nm diameter ± 3.64 and 22.37 nm ± 2.07, respectively (Figure 2a,b). This clearly showed a decrease in particle size due to doping. The particles were not monodispersed and were agglomerated. The EDX analysis of the bare and Mg-doped ZnO NPs showed significant peaks of Mg, Zn, and O, hence confirming the successful doping of Mg in the ZnO crystal lattice (Figure 2c,d).

The results showed reduced ZnO crystallite size after doping with Mg [34] (Table 1).

### 2.5. Distribution of Mg-Doped ZnO NPs within the Composite

**Figure 3:**
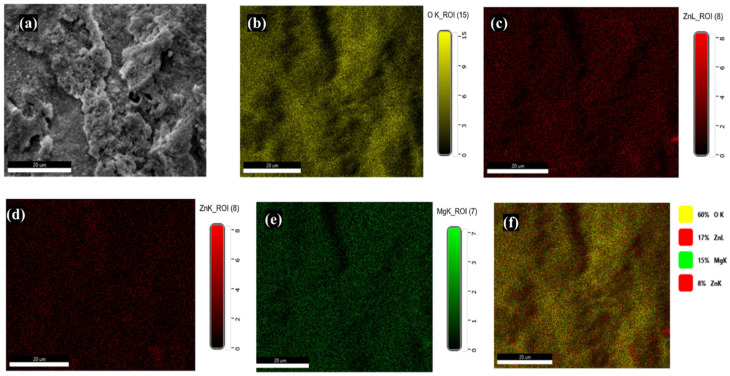
Elemental mapping to confirm the uniform distribution of Mg-doped ZnO NPs in composite (2.5%) at 200 μm (**a**), surface morphology of reinforced composite, (**b**) distribution of Oxygen in reinforced composite, (**c**,**d**) distribution of Zinc in reinforced composite, (**e**) distribution of Magnesium in reinforced composite, and (**f**) overlapped distribution of O, Zn, and Mg.

The morphological studies presented the distribution of Mg-doped ZnO NPs throughout the fabricated composite (Figure 3a–f). In the composite composition map, O, Zn, and Mg appeared in yellow (Figure 3b), red (Figure 3c,d), and green (Figure 3e), respectively. The elemental overlap is shown in Figure 3f.

### 2.6. The Antibacterial Activity of Composites with Bare and Mg-Doped ZnO NPs

**Figure 4:**
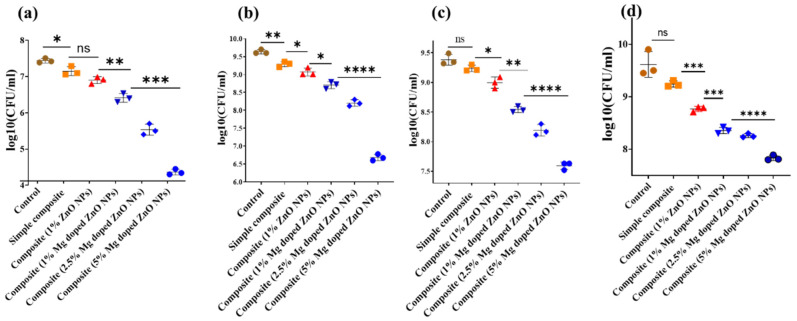
Antibacterial effect of composites with bare and Mg-doped ZnO NPs against (**a**) *S. mutans*, (**b**) *E. faecalis*, (**c**) saliva-derived microcosm, and (**d**) diabetic microcosm. *p*-value < 0.05 is flagged with one star (*), while (**) means *p*-value < 0.01, (***) represents a *p*-value < 0.001 and (****) shows *p* value < 0.0001. ns (not significant) represents statistically insignificance.

The antibacterial activity of composites fabricated with bare and Mg-doped ZnO NPs is presented in Figure 4. Composites with 1% Mg-doped ZnO showed better antibacterial activity than composites with 1% ZnO NPs against *S. Mutans*, *E. faecalis*, and saliva-derived microcosms (*S. mutans*, *p* = 0.0005; *E. faecaclis*, *p* = 0.0074, microcosm, *p* < 0.0001, diabetic microcosm *p* < 0.0001). Enhancing the concentration of Mg-doped ZnO NPs significantly enhanced antibacterial activity; hence, antibacterial activity was concentration-dependent (*S. Mutans*, *p* < 0.0001; *E. faecalis*, *p* < 0.0001, saliva-derived microcosm, *p* < 0.0001).

### 2.7. Compressive Strength of Composites Fabricated with Bare and Mg-Doped ZnO NPs

**Figure 5:**
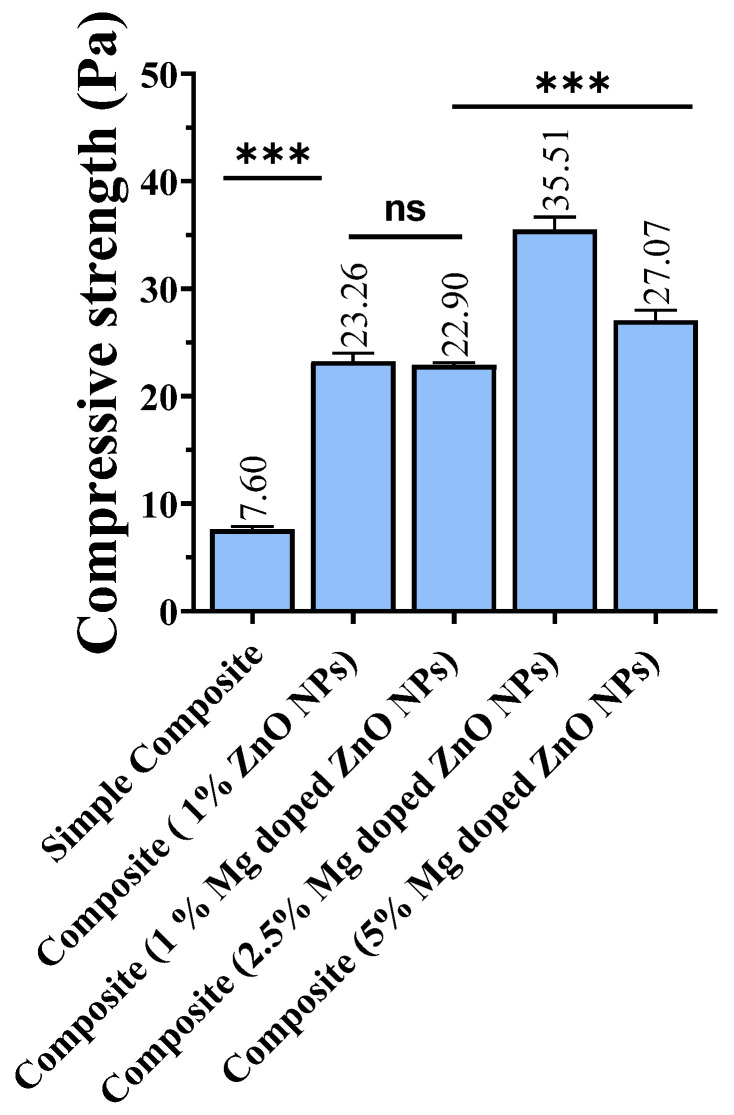
Compressive strength of composites with bare and Mg-doped ZnO NPs at varying concentrations. *p*-value < 0.001 is flagged with three stars (***), while ns (not significant) represents statistically insignificance.

The compressive strength of composites with bare and Mg-doped ZnO NPs is presented in Figure 5. Fabrication of composites with 1% Mg-doped ZnO did not show significant enhancement in the compressive strength when compared with composites with 1% ZnO NPs (*p* = 0.46). However, the compressive strength was better when compared with simple composites (*p* < 0.0001). Enhancing the concentration of Mg-doped ZnO NPs to 2.5% significantly enhanced the compressive strength; nevertheless, a further increase in the concentration of Mg-doped ZnO in composite to 5% significantly reduced the mechanical properties of the resin composite (*p* < 0.0001).

### 2.8. Completion of Polymerization in Composites Fabricated with Bare and Mg-Doped ZnO NPs

The completion of the polymerization of composites with bare and Mg-doped ZnO NPs is stated in Table 2. The difference in the thickness after ethanol shaking was less than 0.3 mm, showing the completion of polymerization for all samples (Table 2). There was a significant decrease in the completion of polymerization with increased concentration of Mg-doped ZnO NPs (*p* = 0.0002).

### 2.9. pH Buffering Effect of Composites Fabricated with Bare and Mg-Doped ZnO NPs

**Figure 6:**
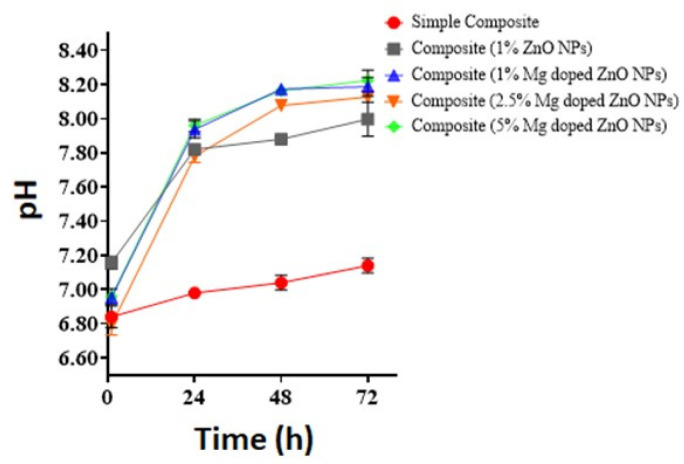
pH buffering effect of composites with bare and Mg-doped ZnO NPs at varying concentrations.

The pH change upon immersion of composites fabricated with bare and Mg-doped ZnO is presented in Figure 6. Fabrication of composites with 1% Mg-doped ZnO NPs showed a significant increase in pH when compared with composites with 1% ZnO NPs at all immersion times. The greatest pH change at all immersion times was observed for composites with 5% Mg-doped ZnO NPs.

### 2.10. Biocompatibility of Composites Reinforced with Bare and Mg-Doped ZnO NPs

**Figure 7:**
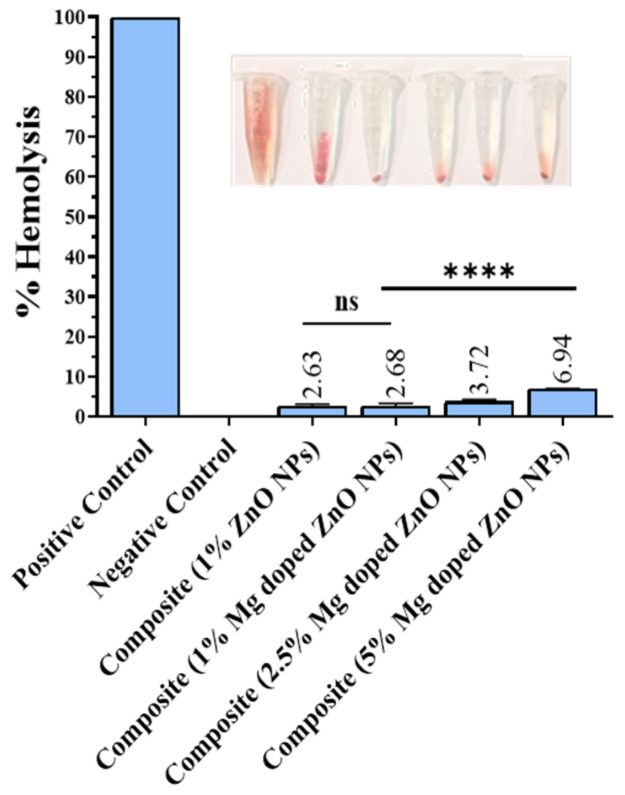
% Hemolysis of composites with bare and Mg-doped ZnO NPs at varying concentrations. *p*-value < 0.0001 is flagged with four stars (****), while ns (not significant) represents statistically insignificance.

The biocompatibility of the composites fabricated with bare and Mg-doped ZnO NPs towards human cells analyzed through hemolysis assay are presented in Figure 7. There was no effect on the % hemolysis upon adding 1% Mg-doped ZnO compared to composites with 1% ZnO NPs (*p* = 0.929). Increasing the concentration of Mg-doped ZnO significantly enhanced hemolysis (*p* ≤ 0.0001). % Hemolysis for all experimental composites came under a safe limit, i.e., a maximum of 5% according to ISO/TR 7406 [37].

### 2.11. Secondary Caries Assessment in the Alloxan-induced Diabetes Model

**Figure 8:**
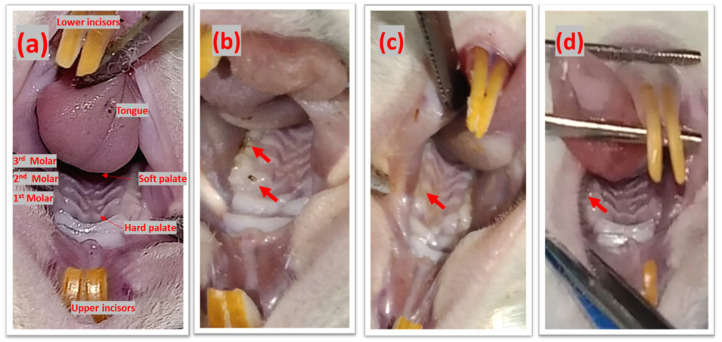
(**a**) Mouth oral cavity, secondary caries assessment, red arrows showing (**b**) marginal caries adjacent to simple composite, (**c**) visual changes in the enamel around the composite with ZnO, and (**d**) sound tooth surface around the restoration composite with Mg-doped ZnO.

Secondary caries assessment was conducted using the CARS tool (Figure 8). Based on visual assessment, in the simple composite group, two out of three rats developed marginal caries adjacent to restoration with underlying dark shadows, whereas one rat developed a carious defect (Figure 8b). In the composite-ZnO group, all three rats developed distinct visual changes in enamel around the restoration margin (Figure 8c). Whereas in the composite-Mg-ZnO group, sound tooth surface with restoration was observed (Figure 8d).

## 3. Discussion

The present study reported the successful synthesis of Mg-doped ZnO and its application as an effective antibacterial agent that retains the mechanical properties and aesthetics of the resin composite. The enhanced antibacterial activity was due to the doping of ZnO with Mg ions [36]. Mg was utilized as a doping agent owing to its mechanical properties, aesthetics, and role in regulating acidic pH [28]. In total, 5% of the doping agent with respect to the Zinc source, that is, zinc acetate, was utilized for doping as it has been reported to increase surface area to volume ratio and decrease crystal size in previous studies [34,35,36,37]. In UV analysis, absorption was enhanced after doping the ZnO with 5% Mg, which confirmed the doping of Mg in the ZnO crystal lattice [35]. This increase in absorption might be due to multiple factors such as oxygen deficiency, particle size, defects in the lattice structure, etc. [36]. The XRD patterns observed for bare and Mg-doped ZnO confirmed the hexagonal crystal shape, as reported in the previous studies [34,35,36,37]. After doping, the intensity of the XRD peak decreased, showing crystallinity loss due to lattice distortion [38]. The EDX studies showed no impurity displaying high purity of the samples. Furthermore, the Mg-doped ZnO NPs were uniformly distributed in the composite to ensure a large interfacial area between the NPs and the constituents of the resin composite [38,39].

After successful synthesis, the Mg-doped ZnO NPs were added to the commercial resin composites, showing no change in the aesthetic properties. The composites with 1% Mg-doped ZnO NPs showed enhanced antibacterial activity compared to composites with 1% ZnO NPs for *S. mutans*, *E. faecalis*, and against saliva-derived microcosm. A similar trend was observed in a previous study where Silver (Ag)-doped ZnO was added to resin composites and showed antibacterial effects against *S. mutans* and saliva microcosm [23]. The antibacterial activity of composites was enhanced by increasing the concentrations of Mg-doped ZnO. A similar trend was observed for the addition of ZnO in the resin composites in a previous study [10]. Antibacterial activity was also observed against diabetic saliva microcosm. The diabetic saliva microcosm differs from normal saliva, as it has higher numbers of culturable acidogenic bacteria such as streptococci and lactobacilli [5,7]. The composites with Mg-doped ZnO also showed enhanced antibacterial activity against diabetic saliva microcosm at all concentrations.

Mechanical properties were enhanced upon adding Mg-doped ZnO compared to simple composites. However, doping did not affect the mechanical properties compared with composites with ZnO. Mechanical properties were enhanced at 2.5 percent and reduced at 5% concentration. The ameliorated compressive strength of the composite at low NPs concentrations can be attributed to enhanced dispersion [21]. In contrast, at higher concentrations, aggregates and agglomerate formation may lead to defects that will eventually deteriorate the mechanical properties of the composite [22]. The completion of polymerization was assessed to look for the effect of the incorporation of Mg-doped ZnO NPs on the polymerization of the resin composite. The incomplete polymerization of composite resins at higher NPs concentrations of Mg-doped ZnO was observed. Ineffective polymerization leads to increased release of residual monomers that may negatively influence clinical performance and promote surface staining and the likelihood of marginal leakage [39,40]. The present study reported that the incorporation of Mg-doped ZnO NPs did not negatively affect the polymerization except at 5% concentration.

For human use, less than 5% hemolysis of a material is considered safe, according to ISO/TR 7406 [37]. The present biocompatibility studies revealed that composites with lower concentrations of Mg-doped ZnO NPs (1 and 2.5%) are safe and nontoxic to human cells. However, they are not safe at concentrations of 5% or more than 5%. The results of the pH change moving towards basic pH with time revealed the pH buffering effect of Mg-doped ZnO, controlling pH around the composites, hence not promoting the growth of caries-producing bacteria. The increase in the pH was due to the release of Mg and Zn ions into the media [31]. As Mg ions are dissolved in the medium, they produce hydrogen and magnesium hydroxide Mg(OH)_2_ and Zn(OH)_2_ increasing the pH and shifting it towards the physiological pH [30,31,32,33]. This may prevent biofilm formation and limit demineralization of the tooth at the enamel-composite interface.

For in vivo evaluation, a modified version of the previously reported rodent model of secondary caries was developed [33,41]. The resin composites were tested on this model to realize the caries-inhibiting effect of composites with Mg-doped ZnO NPs. We modified this model by inducing diabetes in rats after filling operations. The aim was to provide a favorable environment for the growth of caries-causing bacteria and mimicking diabetic oral conditions; the acidic pH of saliva in diabetes limits its acid-buffering effect and promotes the growth of caries-causing bacteria [42].

CARS criteria were utilized in this study, which were not previously utilized to score SC in rat models [43]. It is a validated method of scoring caries in human subjects [43]. According to CARS criteria, the control group (two out of three) scored 4, i.e., formation of marginal caries around restoration with underlying dark shadows, hence showing successful caries formation in the rats. Carious defects were formed within the short period due to inoculated biofilm-making bacteria, i.e., *S. mutans* and *E. faecalis*, and the caries favorable oral environment was created due to diabetes induction [5,6,7,8]. The composites with Mg-doped ZnO NPs showed intact surfaces, whereas composites with simple ZnO NPs exhibited a score of 2, which is a distinct visual change around the composite. This showed that composites with Mg-doped ZnO NPs at 2.5% concentration could be an effective antibacterial resin composite.

Antibacterial activity and prevention of secondary caries in composites with Mg-doped ZnO NPs are attributable to the release of Zn and Mg ions. These positively charged Zn^2+^ and Mg^2+^ released by composites interact with bacterial cell membranes having negative charge through electrostatic interaction and create a transmembrane pore in the cell membrane. This leads to disrupted membrane permeability. These ions penetrate the cell membrane, interact with the sulfhydryl (-SH) group, and suppress enzymes. This negatively affects the respiratory chain and cell division, which increases ROS generation, finally leading to the death of the bacteria [44]. Furthermore, the formation of Mg[OH]_2_ and Zn[OH]_2_ leads to a pH buffering effect, hence, limiting the biofilm formation and prevention of caries [29,30,31,32,33].

### Limitations

There are some limitations of the study which need to be addressed: (i) animal handling was difficult, due to the very small size of the rat teeth; (ii) releases kinetics of Zn and Mg ions from the composites were not explored; (iii) the thermal stability of composites has not been conducted; (iv) a polymer coating of ZnO should have been carried out to prevent aggregation and enhance blending into the composites.

## 4. Materials and Methods

All the chemicals utilized in the present study were procured from Sigma Aldrich (St. Louis, MO, USA) unless indicated.

### 4.1. Synthesis of Bare ZnO NPs

Bare ZnO NPs were prepared by adding Zinc acetate solution (0.01 M) dropwise into the sodium carbonate solution (0.01 M), followed by half an hour of stirring [45]. The white precipitates obtained as a result were centrifuged, followed by the replacement of the reaction medium with deionized water after each centrifugation. Bath sonication (15 min at 23 °C) was performed before each water replacement to suspend the precipitates [46]. The washed precipitates were dried at 80 °C, followed by calcination at 300 °C for 2 h.

### 4.2. Synthesis of Mg-Doped ZnO NPs

For Mg doping, certain amounts of (Magnesium acetate (5% of zinc acetate dihydrate) were dissolved in deionized water under vigorous stirring and added dropwise to M zinc acetate solution (0.01 M). It was then added dropwise to the sodium carbonate solution (0.01 M) under vigorous stirring for 30 min. The resultant precipitate was ultrasonically washed and dried at 80 °C and then calcinated at 300 °C for 2 h [46].

### 4.3. Characterizations of Bare and Mg-Doped ZnO NPs

Multiple characterization techniques were utilized to confirm the synthesis of bare and Mg-doped ZnO NPs. UV–Vis spectrophotometry (UV-2800, BMS Biotechnology Medical Services, Madrid, Spain), scanning electron microscopy (SEM), and energy-dispersive X-ray spectroscopy (EDS) analyses were conducted using the Nova NanoSEM 450 field-emission scanning electron microscope (FE-SEM) (Thermofisher, Waltham, MA, USA). X-ray diffraction (XRD) analysis was carried out using an X-ray diffraction machine S/N 65022 (STOE, Darmstadt, Germany). Fourier-transform infrared spectroscopy (FTIR) analyses were carried out using a Bruker FTIR Spectrometer ALPHA II (Westborough, MA, USA) [46,47,48,49].

### 4.4. Composite Disk Reinforcement

Commercial composite resin, i.e., Nexcomp-META BIOMED containing Bis-GMA, UDMA, and Bis-EMA, along with barium aluminum silicate fillers, were utilized in this study. Bare and doped NPs (varying concentrations, Table 3) were mixed with composite resin by mixing for 60 sec [50]. The cured disks were prepared using a mold (2 mm × 4 mm) following the manufacturer’s instructions. After curing, disks were immersed in deionized water and agitated for 5 min to remove uncured monomers [51]. This was carried out to ensure that antibacterial activity does not occur due to the release of uncured monomers. The disks were finally sterilized with ethanol.

### 4.5. Distribution of Mg-Doped ZnO NPs within Resin Composite

The NPs distribution was mapped within the resin composite by dispersive X-ray microanalysis (EDX) coupled with SEM. The elemental atoms were mapped to decipher NPs within the composite.

### 4.6. Bacterial Isolation

Saliva was collected from 10 volunteers after obtaining their informed consent. The volunteers were selected based on those who had neither chronic illness nor received any antibiotic therapy in the last 6 weeks. The volunteers refrained from food/drink intake for 2 h before the saliva collection. Equivalent amounts of saliva were obtained and vortexed for 60 sec to disperse. Saliva was then diluted in a 1:9 ratio with distilled water, and 50 µL were dropped on Tryptic Soy Agar (TSA) media plates and anaerobically incubated (37 °C, 48 h). Based on colony morphology, the colonies were identified after the incubation period [52,53]. The typical colonies from each plate were streaked on blood agar plates incubated anaerobically (37 °C, 48 h). The overnight cultures were stored in glycerol stock at −80 °C.

### 4.7. Antibacterial in Vitro Assay

In total, 5 mL of tryptic soy broth (TSB) was inoculated separately with *S. mutans* and *E. faecalis* culture, followed by overnight incubation at 37 °C. 5 mL of TSB (with 1% sucrose) broth with 200 µL of preculture. After approximately 3.5 h, when the optical density (OD) of the culture reached 1 at 600 nm, the culture was diluted through serial dilutions and spread over (50 µL) the TSA plate to find the initial number of colonies. Then, 500 µL of the diluted culture for each bacterial strain were added to sterilized microcentrifuge tubes, labeled, and placed inside the well of the microcentrifuge tube-based apparatus. The apparatus was then placed in an incubator and incubated with the composite specimens for 6 h. After 6 h, the disks were removed from the culture and discarded. From each microcentrifuge tube, 50 µL of culture were plated on the TSA plates, and colony-forming units were calculated [4,49].

For normal saliva-derived microcosm, the saliva collected during bacterial isolation was utilized, and the same procedure as above was used for the antibacterial assay [4]. For the diabetic microcosm, the saliva was collected from the volunteers after informed consent was obtained, and the same antibacterial assay was performed. The experiments were performed in triplicate.

### 4.8. Mechanical Testing

Compressive strength was determined to evaluate the influence of varying concentrations of Mg-doped ZnO NPs on the mechanical properties of the resin composite. Initially, the composite disks were immersed in saliva for 24 h. The disks were then removed and dried, followed by an evaluation of compressive strength in a universal testing machine (Shimaszo, Tokyo, Japan) at a crosshead speed of 0.5 cm/min and load cell of 5 KN. Each sample was positioned at the base of the testing machine, and stress was applied until the composite sample was fractured. The compressive strength of the composites was then calculated using the below-provided formula [54].

$$Compressive\;strength\;\left(\mathrm{MPa}\right)=\frac{Failure\;load\;of\;the\;\mathrm{experimental}\;composite\;\left(N\right)}{Area\;of\;the\;\mathrm{experimental}\;composite\;\left(\mathrm{mm}2\right)}.$$

### 4.9. Completion of Polymerization in Composites Reinforced with Mg-Doped ZnO NPs

The ethanol shaking test was conducted to measure the completion of polymerization after curing. The composite resin specimens, 2 mm in width and 4 mm in diameter, were light-cured. Each composite disk was placed in the microcentrifuge tube containing 1 mL of ethanol. It was then agitated for 20 sec using a vortex. The thickness of the composite was again measured, and the difference in the thickness values was measured to see if the polymerization of each sample was complete [55].

### 4.10. pH Evaluation

A previously reported pH assay was performed to assess the pH buffering effect of experimental composite disks [38,39]. The prepared composite disks were immersed in plastic vials with 5 mL of deionized water (pH 6.6). The plastic vials were then sealed, followed by incubation at 37 °C. The pH was measured using a calibrated pH-meter EcoMet P25 (Isteck, Seoul, Republic of Korea) after 1, 24, and 72 h of immersion. Before each measurement, vials were shaken for 5 sec to ensure that released ions were uniformly distributed [56].

### 4.11. Biocompatibility of Composites with Mg-doped ZnO NPs

A hemolysis assay was conducted to assess the biocompatibility of the composites with varying concentrations of Mg-doped ZnO. After receiving informed consent, blood was collected from a healthy female volunteer. The collected blood was centrifuged at 18,000× *g* rpm for 10 min to collect red blood cells (RBCs). The RBCs were diluted with phosphate-buffered saline (PBS) (1:3 ratio). Test tubes with 10 mL PBS solution were prepared, and composite samples were placed in them and incubated in a water bath (15 min, 37 °C). Approximately 200 µL of diluted RBCs were then added to the tubes with composites, gently inverted to ensure homogenous suspension, and incubated for 2 h. Triton X-100 (1%) and PBS were used as the positive and negative control, respectively. After incubation, the composites were removed, and blood samples were centrifuged (18,000× *g* rpm) for 10 min to obtain supernatant. The optical density (OD) was measured at 350 nm wavelength, and percentage hemolysis was calculated using the below-provided formula [37].

$$Hemolysis\;\left(\%\right)=\frac{\left(OD\;of\;sample\;at\;350\;\mathrm{nm}-OD\;of\;negative\;control\;at\;350\;\mathrm{nm}\right)}{\left(OD\;of\;positive\;control\;at\;350\;\mathrm{nm}-OD\;of\;negative\;control\;at\;350\;\mathrm{nm}\right)}.$$

### 4.12. In Vivo Studies

**Figure 9:**
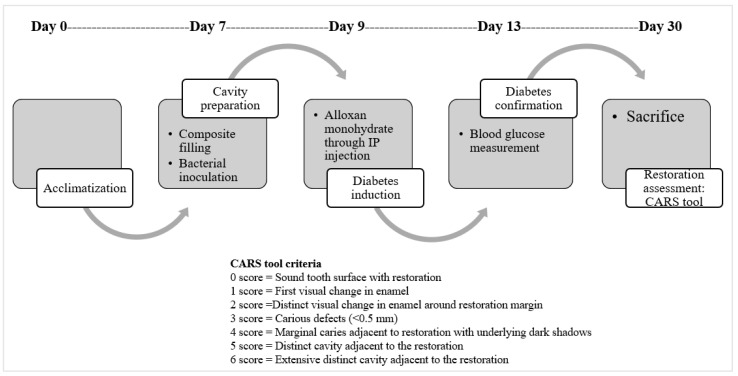
Secondary caries assessment in the diabetic rodent model.

In vivo testing of composites was conducted in an alloxan-induced diabetes model; the model was developed by modifying a previously reported rat model of secondary caries [41]. Female Wistar rats (*n* = 9) (National Institute of Health (NIH), Islamabad, Pakistan) were obtained at the age of 8 weeks. They were kept at 27 ± 2 °C, under a light–dark cycle, and provided with tap water and a normal diet ad libitum. The rats were then acclimatized for 7 days, followed by grouping into three cages (simple composite group (*n* = 3), ZnO-composite group (*n* = 3), and Mg-ZnO-composite group (*n* = 3)). On day 7, rats were anesthetized and placed in a dorsal position for cavity preparation and filling procedure. In the maxillary first and second molars, cavities were prepared (0.3–0.5 mm depth), and filling operations were conducted according to the manufacturer’s protocol. After this, *S. mutans* and *E*. *Faecalis* (10^8^ CFU/mL, 0.3 mL) were inoculated into the rat’s mouth twice at a time interval of 30 min for two consecutive days. During this period, rats were provided with a cariogenic diet and 5% sucrose water. On the 9th day, diabetes was induced in the rats through intraperitoneal injections of alloxan monohydrate (150 mg/Kg) [32]. After 3 weeks, the rats were sacrificed, and restoration assessments were carried out using the CARS criteria based on the “Caries Associated with Restorations or Sealants” (CARS) criteria described by the International Caries Classification and Management System [43] (Figure 1). The restoration surfaces were evaluated with a 0 (‘sound tooth surface with restoration’) to 6 (‘extensive distinct cavity with visible dentin’) score (Figure 9) [43].

### 4.13. Statistical Analysis

Statistical analysis was conducted using GraphPad Prism (Version 9, San Diego, CA, USA). Mean and standard deviations were reported. Furthermore, group comparisons were carried out using a *t*-test and one-way ANOVA analysis.

## 5. Conclusions

This study reported the effectiveness of Mg-doped ZnO NPs as an antibacterial agent in dental resin composite restorative materials to prevent secondary caries. Mg-doped ZnO NPs (up to 2.5% (*w*/*w*)) enhanced the antibacterial properties of the resin composites without compromising the mechanical properties, aesthetics, and biocompatibility. Furthermore, the pH buffering effect did not allow favorable conditions for biofilm formation. The composites with Mg-doped ZnO NPs also successfully prevented secondary caries in the diabetic rodent model compared to simple composites. Hence, composites with Mg-doped ZnO can be an effective candidate for the treatment of caries even in high-risk diabetic patients. Further studies must focus on ZnO doped with other functional materials.

## Figures and Tables

**Table 1:** The particle size of bare and Mg-doped ZnO NPs using X-ray diffraction data.

		2 theta	FWHM	d	Size = D (nm)
1	ZnO NPs	35.78	0.321	2.517	26.0060054
2	Mg-doped ZnO NPs	35.407	0.481	2.533	17.3372344

**Table 2:** The difference in width of composites after ethanol shaking test.

Sample	Mean Width after Ethanol Shaking (D2)	Standard Deviation (SD)	DI-D2 (Width before–Width after Ethanol Shaking)
Simple composite	1.91	0.01	0.09
Composite (1% ZnO NPs)	1.933	0.005	0.067
Composite (1% Mg-doped ZnO NPs)	1.930	0.02	0.07
Composite (2.5% Mg-doped ZnO NPs)	1.88	0.03	0.12
Composite (5% Mg-doped ZnO NPs)	1.75	0.02	0.25

**Table 3:** Chemical composition of experimental composites.

Sample	Matrix Monomers	Fillers (% by Weight)	Antibacterial Agent
Simple composite	UDMA, Bis-GMA, Bis-EMA (40:40:20 ratio)	Barium aluminum silicate 75%	-
Composite (1% ZnO NPs)	UDMA, Bis-GMA, Bis-EMA (40:40:20 ratio)	Barium aluminum silicate 75%	1% ZnO NPs
Composite (1% Mg-doped ZnO NPs)	UDMA, Bis-GMA, Bis-EMA (40:40:20 ratio)	Barium aluminum silicate 75%	1% Mg-doped ZnO NPs
Composite (2.5% Mg-doped ZnO NPs)	UDMA, Bis-GMA, Bis-EMA (40:40:20 ratio)	Barium aluminum silicate 75%	2.5% Mg-doped ZnO NPs
Composite (5% Mg-doped ZnO NPs)	UDMA, Bis-GMA, Bis-EMA (40:40:20 ratio)	Barium aluminum silicate 75%	5% Mg-doped ZnO NPs

## Data Availability

Not applicable.
